# Giant serous cystadenoma arising from an accessory ovary in a morbidly obese 11-year-old girl: a case report

**DOI:** 10.1186/1752-1947-2-7

**Published:** 2008-01-18

**Authors:** Steven M Sharatz, Taína A Treviño, Luís Rodriguez, Jared H West

**Affiliations:** 1 Department of Obstetrics and Gynecology, Ponce School of Medicine, PO Box 7004, Ponce, PR 00732-7004, Puerto Rico

## Abstract

**Introduction:**

Ectopic ovarian tissue is an unusual entity, especially if it is an isolated finding thought to be of embryological origin.

**Case presentation:**

An 11-year-old, morbidly obese female presented with left flank pain, nausea, and irregular menses. Various diagnostic procedures suggested a large ovarian cyst, and surgical resection was performed.

**Conclusion:**

Histologically, the resected mass was not of tubal origin as suspected, but a serous cystadenoma arising from ovarian tissue. The patient's two normal, eutopic ovaries were completely uninvolved and unaffected. A tumor arising from ectopic ovarian tissue of embryological origin seems the most likely explanation. We suggest refining the descriptive nomenclature so as to more precisely characterize the various presentations of ovarian ectopia.

## Introduction

Ectopic ovarian tissue is a rare phenomenon, with an incidence estimated between 1 in 29,000 and 1 in 700,000 gynecologic admissions. A more accurate estimate is difficult due to a confusing and still disputed classification system, as well as the frequently asymptomatic nature of the condition. We report a case of what is best described as a giant serous cystadenoma arising from an accessory ovary in a morbidly obese 11-year-old girl.

## Case presentation

An 11-year-old girl presented with two bouts of abdominal and left flank pain during a 5-month period, described as non-radiating and 8 out of 10 in intensity. The pain was accompanied by nausea and one episode of vomiting. The patient also noticed a decrease in urinary frequency during the same interval. She denied fever, dysuria, hematuria, or bloody stools. Past medical and family history was unremarkable. The patient had no history of hospitalizations, surgeries, or chronic illness. Menarche was at the age of 10 followed by irregular cycles, occurring every 40 to 50 days with very heavy flow.

**Figure 1:**
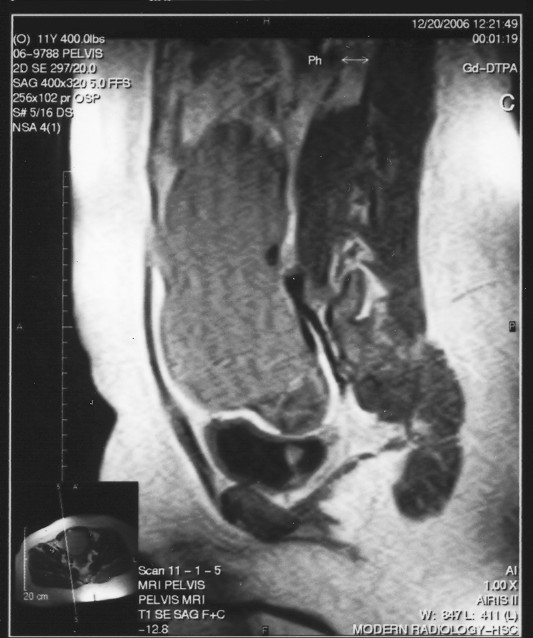
Sagittal T1-weighted MRI. A large, fluid-density, multilobular cystic structure is seen roughly at the midline and extending to the level of the umbilicus. Although the cyst appears to originate on the right, it was discovered at laparotomy to be attached to the left fallopian tube.

**Figure 2:**
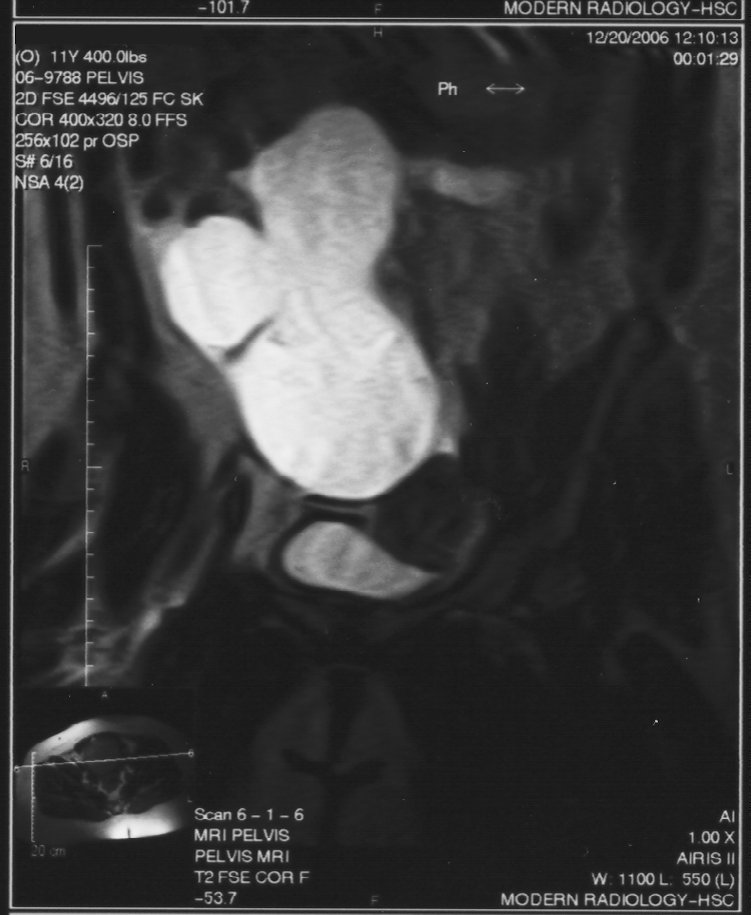
Coronal T2-weighted MRI.

Physical examination revealed a morbidly obese (weight: 232 lbs., BMI: 42) adolescent girl. Her abdomen was soft and depressible and no masses were identified on palpation. Various imaging studies were performed including a pelvic ultrasound, which identified an 18.7 cm × 10.0 cm × 15.4 cm cystic lesion that extended into the abdomen to about the level of the umbilicus. Two MRI studies were ordered which identified a large cystic structure that appeared to originate from the *right *adnexa, suggesting an ovarian tumor [Figures 1, 2]. Tumor markers were measured (CA-125: 30.2 U/ml, CA-19-9: 18 U/ml, AFP: 3 U/ml, LDH: 543 U/ml), and were all within normal limits. A quantitative hCG and pregnancy test were negative. A presumptive diagnosis of ovarian cyst was made.

At laparotomy the cyst was found attached to the fimbriated end of the *left *fallopian tube, with which it shared its blood supply. On gross inspection, it was a smooth, multilobulated, fluid-filled mass with no attachments to the left ovary itself. On close examination, the patient's two ovaries showed no signs of torsion or necrosis: both were smooth and atraumatic. Due to the size and location of the cyst, a left salpingectomy was performed in order to remove it completely. The patient was left with two intact ovaries and her right fallopian tube. The presence of the two normally situated ovaries was documented on follow-up sonogram.

**Figure 3:**
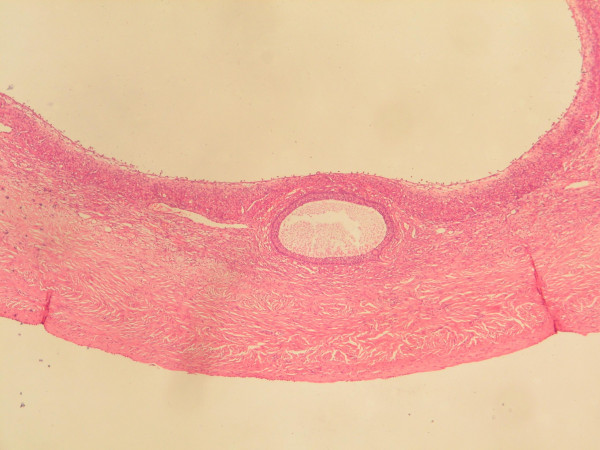
Histology of the resected mass shows a Graafian follicle and an inner lining of ciliated columnar epithelium, consistent with a benign cystadenoma derived from ovarian tissue.

Due to the identification of two eutopic ovaries and the attachment to the mass to the left fallopian tube, a post-operative presumptive diagnosis of a left paratubal cyst was made. On histological examination the specimen was shown to be lined by columnar epithelial cells with abundant cilia, and contain primary follicles, corpora albicans, Graafian follicles, and areas of fibrin deposition [Figure 3]. The final histopathological diagnosis was hemorrhagic serous cystadenoma arising from ovarian tissue. Patient has recovered uneventfully from the procedure.

## Discussion

We are aware of at least 50 published reports of additional ovarian tissue since Wharton published his seminal description in 1959 [1]. He defined an *accessory *ovary as having close proximity and some form of association to a eutopic ovary and its associated blood supply. The term *supernumerary *ovary was reserved for ovarian tissue far removed from the eutopic ovaries and with a separate blood supply. The former is often found attached to the fallopian tube or one of the various ligamentous structures of the ovarian-uterine complex; the latter can be found anywhere along the embryological migratory path of the ovarian primordium, including the mesentery, retroperitoneal space, and omentum [2].

The terminology employed has caused substantial confusion on the subject. The terms *supernumerary *and *accessory *are somewhat misleading because their definitions by Wharton presuppose two normal ovaries and an embryologic origin for the additional ovarian tissue. It has been suggested that up to 50% of cases of additional ovaries are actually post-inflammatory or post-surgical implants [3,4]. Lachman et al have suggested doing away with the traditional terms and labeling all abnormally placed ovarian tissue as *ectopic*, subcategorized as either post-surgical, post-inflammatory, or truly embryological [3]. Unfortunately, this schema fails to make a distinction between 1) extra tissue that is present *in addition *to two eutopic ovaries and 2) that which exists *in place of *a eutopic ovary because it is the result of defective migration or development of an ovarian primordium [5]. Therefore, it is difficult to precisely determine the incidence and categorize the characteristics of the phenomenon.

About 36% of reported cases of ectopic ovary are associated with urogenital anomalies [6]. Their incidence in patients with absent uterus is as high as 20%, and in as many as 42% of cases of unicornuate uterus there is associated ectopia, and often malformation, of the ovary contralateral to the developed cornu [7]. The majority of cases are classified as *supernumerary *by the Wharton criteria. The detection of both supernumerary and accessory ovaries is often associated with tumors or cysts, perhaps precisely because these are symptomatic and require subsequent workup. Some authors support the idea that this association is due to increased pathological potential of the ectopic tissue [6].

The most common masses identified are mature teratomas and mucinous cystadenomas, present in up to one fifth of patients [5]. In addition, Brenner's tumor [8], sclerosing stromal tumor [9], serous cystadenoma [10], serous cystadenofibroma [11], fibroma [12], and adenocarcinoma have been described. Common clinical presentations involve abdominal pain and irregular menses.

Despite the strong association with pathological processes, supernumerary and accessory ovarian tissue has been notoriously difficult to diagnose preoperatively. It is usually an incidental finding or a surprise histopathological diagnosis after resection of a clinically relevant mass, as occurred in this case. It can be suspected on the basis of hormonal abnormalities, such as continued cyclic endometriosis pain [4] or intact estrogenic response to human chorionic gonadotropin [13] after bilateral oopherectomy. Fujiwara et al. have even made a presumptive diagnosis based on cyclic, FSH-associated changes in a cystic mass, visualized by ultrasound [14]. Normally, however, the nature of the mass is uncertain until histological confirmation is obtained.

The patient's young age and impressive weight are unusual features of this case. To our knowledge, there have only been five previously reported cases of additional ovaries diagnosed in children under the age of eighteen. This includes the two neonatal diagnoses reported by Kuga et al [2]. If the child's obesity is related somehow to a rapid progression of the tumor that led to the relatively early detection, the mechanism is uncertain: although various hormone and gonadotropin receptors have been detected to varying degrees on samples from the spectrum of serous ovarian neoplasms, they have not been shown definitively to promote tumor growth [15,16]. Unfortunately, we do not have comprehensive hormone levels for our patient, although one would expect her estrogen levels to be increased (due to obesity) and her FSH levels to be chronically decreased (due to pituitary axis inhibition); her ovaries were not polycystic and she was not hirsute, suggesting normal LH and androgen levels.

To improve the precision of the terminology, we would propose that the term *ectopic *continue to refer to any inappropriately placed ovarian tissue, regardless of etiology or the presence of two eutopic ovaries. The description can be fine-tuned according to the salient features of the specific presentation and its suspected etiology, e.g. "extra/additional" if accompanied by normal ovaries, or "malformed" if the product of faulty migration or malformation of a would-be eutopic gonad. One can invoke the term "implant" when that etiology is suspected, and Lachman's proposed adjectives "post-surgical" and "post-inflammatory" applied. All permutations of etiology and location can thus be accurately and completely described (e.g., "ectopic extra ovary," "post-inflammatory ectopic implant," or "unilateral ectopic ovarian malformation/remnant"), not previously possible. The terms *supernumerary *and *accessory *should retain their traditional Whartonian definitions in that they refer to distinct presentations of additional (extra) ovarian tissue.

## Conclusion

Our case represents an accessory ovary according to the Wharton criteria, given its adnexal location and a blood supply continuous with that of the fallopian tube. We believe that the tissue is truly embryologically ectopic, to reference Lachman's nomenclature, because of the absence of previous pelvic or abdominal surgery or disease; also significant is the smooth, atraumatic appearance of the eutopic ovaries at laparotomy. To our knowledge, this is the second report of a serous cystadenoma arising from an accessory or supernumerary ovary, and it is among the largest masses reported arising from either.

## Competing interests

The author(s) declare that they have no competing interests.

## Authors' contributions

All authors have read and approved the final manuscript for publication.

1) SS performed manuscript writing, literature review, and collection and analysis of pertinent clinical information.

2) TT participated in the clinical management of the patient, the surgery in which the sample was removed, collection and analysis of pertinent clinical information, and literature review.

3) LR was the attending physician on the case, and therefore performed the surgery and managed the clinical care of the patient; he also gave the authorization for final publication.

4) JW participated in literature review, and collection and analysis of pertinent clinical information.

## Consent

Written consent was obtained from the patient's legal guardian (mother) for the publication of this case report and any accompanying images. A copy of this written consent is available for review by the Editor-in-Chief of this journal.
